# Immunometabolic and potential tumor-promoting changes in 3D cervical cell models infected with bacterial vaginosis-associated bacteria

**DOI:** 10.1038/s42003-022-03681-6

**Published:** 2022-07-22

**Authors:** Jason D. Maarsingh, Paweł Łaniewski, Melissa M. Herbst-Kralovetz

**Affiliations:** 1 Department of Obstetrics and Gynecology, College of Medicine-Phoenix, University of Arizona, Phoenix, AZ USA; 2 Department of Basic Medical Sciences, College of Medicine-Phoenix, University of Arizona, Phoenix, AZ USA

**Keywords:** Cervical cancer, Cytokines, Bacterial infection, Mechanisms of disease, Metabolomics

## Abstract

Specific bacteria of the human microbiome influence carcinogenesis at diverse anatomical sites. Bacterial vaginosis (BV) is the most common vaginal disorder in premenopausal women that is associated with gynecologic sequelae, including cervical cancer. BV-associated microorganisms, such as *Fusobacterium, Lancefieldella, Peptoniphilus*, and *Porphyromonas* have been associated with gynecologic and other cancers, though the pro-oncogenic mechanisms employed by these bacteria are poorly understood. Here, we integrated a multi-omics approach with our three-dimensional (3-D) cervical epithelial cell culture model to investigate how understudied BV-associated bacteria linked to gynecologic neoplasia influence hallmarks of cancer in vitro*. Lancefieldella parvulum* and *Peptoniphilus lacrimalis* elicited robust proinflammatory responses in 3-D cervical cells. *Fusobacterium nucleatum* and *Fusobacterium gonidiaformans* modulated metabolic hallmarks of cancer corresponding to accumulation of 2-hydroxyglutarate, pro-inflammatory lipids, and signs of oxidative stress and genotoxic hydrogen sulfide. This study provides mechanistic insights into how gynecologic cancer-associated bacteria might facilitate a tumor-promoting microenvironment in the human cervix.

## Abstract (web summary)

Metabolomics of 3D cervical cell cultures infected with bacteria associated with bacterial vaginosis (BV) provides insights into how BV-associated bacteria may facilitate a tumour-promoting microenvironment.

## Introduction

In recent decades, specific bacteria have been shown to employ numerous mechanisms that promote carcinogenesis in a variety of cancers. *Helicobacter pylori*, a primary mediator of stomach cancer, was the first bacterium described to be associated with a specific cancer^1^. *Fusobacterium nucleatum* is often found in the gut and is highly correlated with colorectal cancer^2^. In the oral cavity, *Porphyromonas gingivalis* supports oral neoplasia^3,4^. In addition to specific microorganisms, growing evidence supports a causal role of dysbiosis of the human microbiota in cancer development^5^. Recently, we reviewed the key bacteria linked to gynecologic cancer^6^ and found that mechanistic in vitro studies are needed to elucidate the pathogenic and pro-oncogenic mechanisms employed by these understudied bacterial species in the female reproductive tract (FRT).

Cervicovaginal dysbiosis or bacterial vaginosis (BV) is characterized by depletion of health-associated lactobacilli accompanied by overgrowth of a polymicrobial consortium of anaerobic bacteria^7^. Importantly, BV, the most common genital infection, has been associated with serious gynecologic and obstetric sequelae, such as preterm birth, pelvic inflammatory disease, and cervical cancer^6^. Cervicovaginal dysbiosis and BV confer increased susceptibility to infection with oncogenic HPV genotypes, genital inflammation, and cervical neoplasia^8,9^. Next-generation sequencing of the microbiota in the upper and lower FRT has revealed specific bacteria associated with gynecologic cancers^6^, frequently in the clinical setting of BV or cervicovaginal dysbiosis. Yet, these species are often detected at relatively low levels in the lower FRT. Recent meta-analyses based on both cross-sectional and longitudinal clinical studies have provided links between cervicovaginal dysbiosis, persistent HPV infection, and cervical carcinogenesis^10,11^. Our group and others have demonstrated that bacteria belonging to the order Fusobacteriales (*F. nucleatum, Fusobacterium necrophorum*, and *Sneathia* spp.) are associated with HPV infection, cervical intraepithelial neoplasia, and invasive cervical carcinoma^8,12–14^. Notably, *Sneathia* spp. are emerging pathogens and frequently present in women diagnosed with BV^6,15^. In addition to cervical cancer, two cross-sectional clinical studies have evaluated the microbiome of women with endometrial cancer relative to benign controls. Co-colonization of *Fannyhessea vaginae* (formerly classified as *Atopobium vaginae*) with *Porphyromonas* sp. and *Peptoniphilus* were associated with endometrial cancer^16^. However, these limited studies need to be confirmed and expanded in clinical studies with greater patient enrollment. Despite the strong clinical data linking BV to HPV infection and cervical carcinogenesis, the pathogenic mechanisms exploited by BV-associated bacteria (BVAB) to facilitate a pro-carcinogenic cervicovaginal microenvironment remain poorly understood. By coupling advanced in vitro cell culture models and omics technologies, we can investigate the pro-carcinogenic mechanisms exploited by these BVAB.

In this study, we used our well-characterized and physiologically relevant three-dimensional (3-D) human cervical epithelial cell model based on the rotating-wall vessel technology to investigate host-bacterial interactions and provide the foundation to reveal pro-carcinogenic mechanisms^17–20^. The 3-D human model recapitulates in vivo characteristics of parental tissue, such as apical polarization, expression of Toll-like receptors and mucins, and formation of microvilli and intercellular tight junctions that are absent or weakly present in conventional monolayer cultures^17,18^, thus allowing us to more accurately dissect host-microbe interactions and hallmarks of cancer. Furthermore, 3-D cervical cells generate innate immune responses to microbial products and infection with STI pathogens and BVAB that recapitulate clinical responses and findings^17,19–23^. We hypothesize that BVAB associated with gynecologic cancers induces hallmarks of cancer corresponding to inflammation and oncogenic metabolomes that may create a tumor-promoting microenvironment. We have recently studied the immunometabolic influences of health-associated cervicovaginal bacteria (*Lactobacillus crispatus*) and specific BVAB more frequently associated with BV (*Gardnerella vaginalis*, *Fannyhessea vaginae, Prevotella bivia, Sneathia amnii*, and *Megasphaera*)^22,23^. In this report, we utilized a similar multi-omics approach to investigate inflammatory, metabolic, and tumor-promoting properties of five understudied BVAB isolated from the lower FRT and reported to be associated with inflammation and cancer. This study revealed pro-inflammatory and metabolic changes (hallmarks of cancer) elicited by understudied BVAB that may promote carcinogenesis in the human FRT.

## Results

### BVAB associated with cancer exert species-specific cytotoxicity against cervical cell monolayers and colonize 3-D cervical cells

We first screened BVAB associated with cancer in cervical cell monolayers. Cell monolayer cultures were infected with *Lancefieldella parvula* (formerly classified as *Atopobium parvulum*)*, Fusobacterium gonidiaformans, F. nucleatum, Peptoniphilus lacrimalis*, and *Porphyromonas uenonis* at three doses corresponding to final optical densities at 600 nm (OD_600_) of 0.1, 0.01, and 0.001 per 1 × 10^5^ cervical cells/mL. Using trypan blue exclusion staining, we found that all strains except *L. parvula* induced modest cytotoxicity at all doses tested (Supplementary Fig. 1). *L. parvula* induced the greatest cytotoxicity at the highest infection dose, indicated by a 70% decrease in cervical cell viability. *F. gonidiaformans, F. nucleatum, P. lacrimalis*, and *P. uenonis* induced significant (*p* < 0.05) cytotoxicity at high and medium doses; however, cervical cell viability decreased less than 20% relative to mock-infected (PBS-treated) controls. These data demonstrate that in cervical cell monolayer cultures, *L. parvula* exerts the greatest cytotoxic potential relative to *F. gonidiaformans, F. nucleatum, P. lacrimalis*, and *P. uenonis*.

**Fig. 1:**
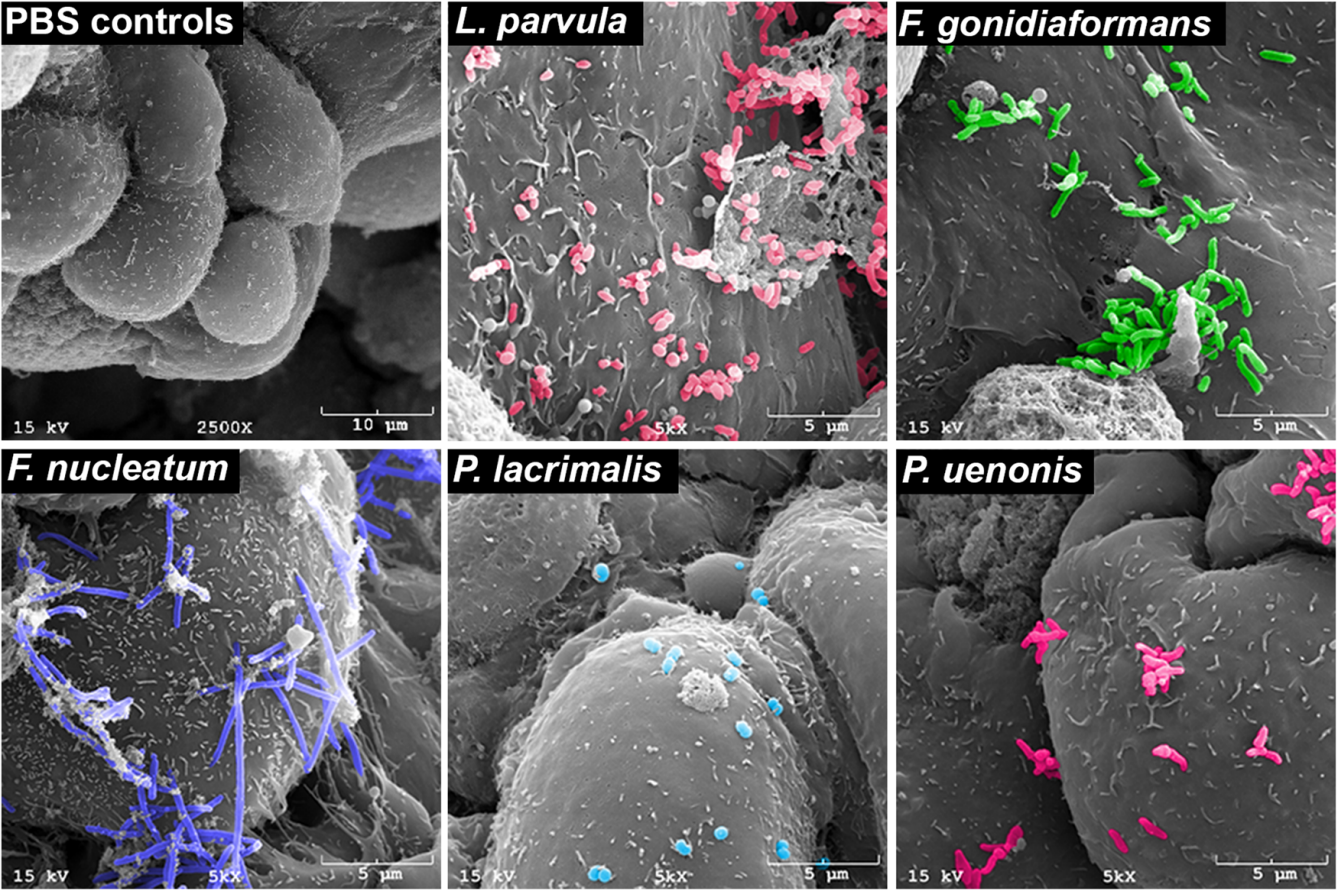
BVAB colonize 3-D cervical cells. Mock-infected 3-D cervical cells (upper left) demonstrating cobblestone appearance and apical polarity. *L. parvula* (upper middle) forms small coccobacilli clusters and colonize healthy cells, as well as necrotic cell material (upper right corner of the micrograph). *F. gonidiaformans* (upper right) colonizes the smooth face of 3-D cervical cells in clusters. *F. nucleatum* (lower left) forms long, filamentous, rugged rod structures that broadly colonize 3-D cervical cells. *P. lacrimalis* (bottom middle) colonizes 3-D cervical cells mostly as pairs or isolated cocci. *P. uenonis* (lower right) appears as short bacilli that colonize the surface and crevices of 3-D cervical cells, often in small clusters. All bacteria were processed in PBS and adjusted to an optical density at 600 nm (OD_600_) of 5.0. Human 3-D cervical cells were infected with bacterial suspensions (20 μl per 1 × 10^5^ epithelial cells) for 4 h under anaerobic conditions and processed for SEM analysis.

We used scanning electron microscopy (SEM) to confirm that tested BVAB (*L. parvula, F. gondiaformans, F. nucleatum, P. lacrimalis*, and *P. uenonis*) colonize 3-D cervical epithelial cells (Fig. 1). Mock-infected (PBS-treated) 3-D cervical cells were also imaged and displayed a characteristic cobblestone appearance, apical polarity, and were covered with microvilli, as previously described^17^. All BVAB tested colonized 3-D cervical cells, although relative abundances and spatial adherence differed between species. *L. parvula* colonized the 3-D cervical cells in small clusters and were often found to be associated with necrotic cellular debris in multiple microscopy fields and micrographs. *F. gonidiaformans* adhered to healthy cells in close proximity to necrotic cell debris. *F. nucleatum* also efficiently colonized 3-D cervical cells and formed long filamentous structures occasionally associated with extracellular material. *P. lacrimalis* colonized 3-D cervical cells as dispersed cocci and were found as isolated or diploid cocci. *P. uenonis* colonized the surface and crevices between 3-D cervical cells to an intermediate degree relative to other BVAB tested and were mostly found in small clusters in multiple fields and images (representative micrograph shown). The SEM analysis confirmed that the BVAB tested in this study colonized 3-D cervical cells in species-specific patterns.

### BVAB associated with cancer induces pro-inflammatory or anti-chemotactic responses in 3-D cervical cells and modulate tumor biomarkers associated with apoptosis

**Fig. 2:**
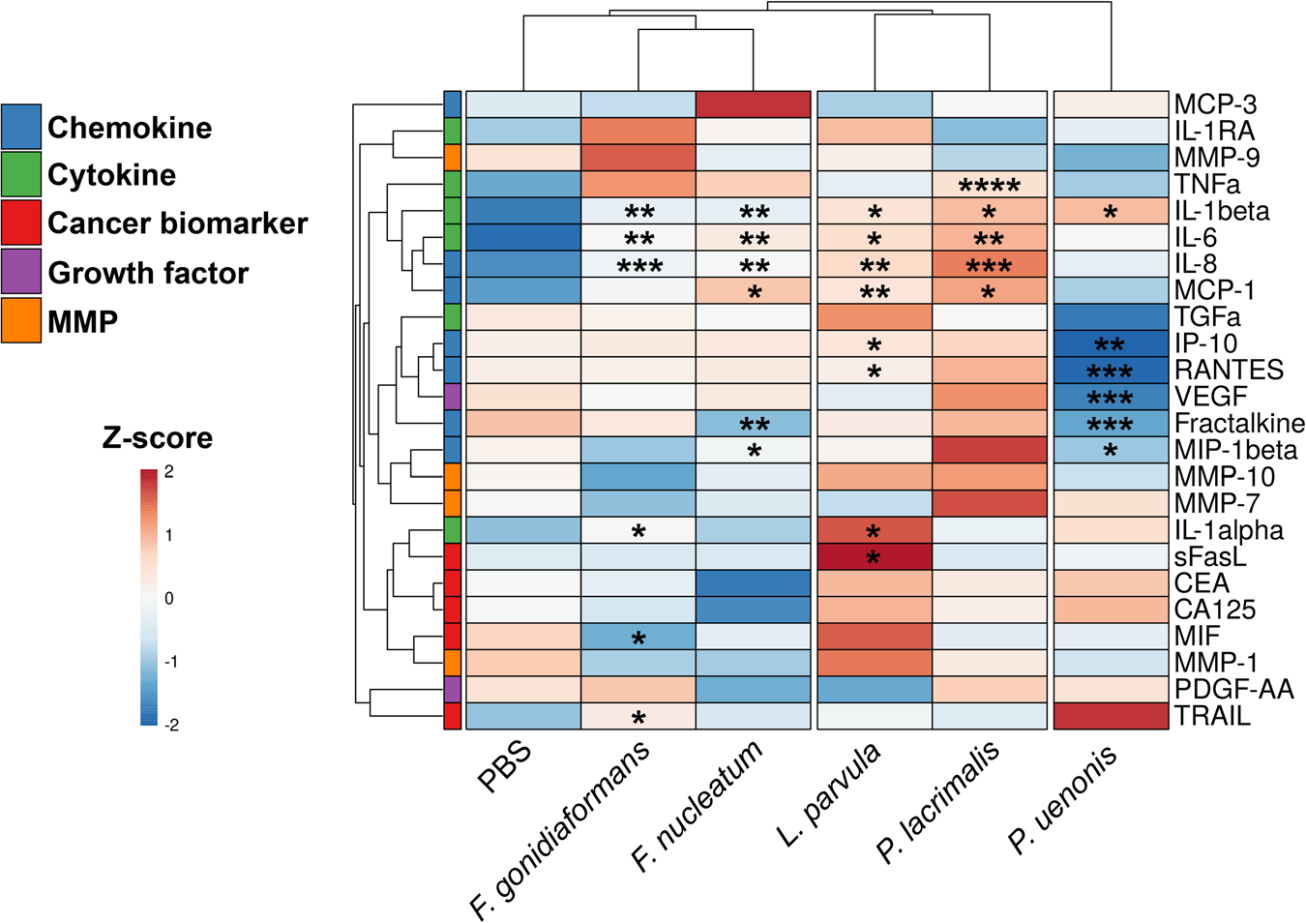
*P. lacrimalis* and *L. parvula* elicits 3-D cervical cell pro-inflammatory responses while *P. uenonis* dampens the chemotactic response, possibly to evade immune clearance. Heatmaps displaying relative concentrations of 3-D human cervical cell secretion profiles of cytokines, chemokines, growth factors, cancer biomarkers, and matrix metalloproteinases. Hierarchical clustering of immunoproteomic targets (rows) and treatments (columns) were calculated using Euclidean distance measures and average linkage clustering algorithms. The data were log-transformed and autoscaled prior to clustering. *p < 0.05; **p < 0.01; ***p < 0.001; ****p < 0.0001; unpaired two-tailed Student’s *t*-test (infection vs. mock-infected controls). All bacteria were processed in PBS and adjusted to an optical density at 600 nm (OD_600_) of 0.5. Human 3-D cervical cells were infected with bacterial suspensions (20 μl per 1 × 10^5^ epithelial cells) for 24 h under anaerobic conditions. A minimum of *n* = 3 independent replicates were performed and measured for each condition with two technical replicates measured within each condition. Cell culture supernatants were used for Bio-Plex analysis.

To evaluate the immunomodulatory potential of BVAB associated with gynecologic cancers, we used cytometric bead arrays to measure 15 secreted proteins corresponding to cytokines, chemokines, and growth factors in culture supernatants of 3-D cervical cells infected with *L. parvula, F. gonidiaformans, F. nucleatum, P. lacrimalis*, or *P. uenonis*. Hierarchical clustering analysis (HCA) of immunoproteomic profiles demonstrated that *F. gonidiaformans* and *F. nucleatum* grouped closely together (Fig. 2), suggesting a shared mechanism of immunomodulatory action within the *Fusobacterium* genus; however, both *Fusobacterium* species also shared a parent cluster with mock-infected controls, indicating a relatively modest immunomodulatory potential by these species. *P. uenonis* clustered separately from all other infections (Fig. 2) indicating this species elicits unique immune responses from 3-D cervical cells relative to all other BVAB tested. *L. parvula* and *P. lacrimalis* clustered together and induced the greatest pro-inflammatory response relative to all tested BVAB. The immunoproteomic profiles also demonstrated that BVAB associated with gynecologic cancers share a core pro-inflammatory response mediated through upregulated expression of IL-1β. All species except *P. uenonis* upregulated the expression of IL-6 and IL-8.

In contrast, *P. uenonis* dampened pro-inflammatory and chemotactic responses, which may promote evasion of clearance by innate and adaptive immune cells. Infection with *P. uenonis* significantly (*p* < 0.05) downregulated the expression of four chemokines: interferon-inducible protein-10 (IP-10), macrophage inflammatory protein-1β (MIP-1β), fractalkine, regulated upon activation, normal T cell expressed and secreted (RANTES), and vascular endothelial growth factor (VEGF) (Fig. 2 and Supplementary Fig. 2). These results demonstrate that, when compared to other tested BVAB species, *P. uenonis* suppresses chemotactic cytokine expression in 3-D cervical cells, which may serve to promote chronic infection.

The genera and species tested in this study has been previously associated with gynecologic cancers^12,16,24^, thus we quantify soluble cancer biomarkers (known to be produced by the A2 cervical cell line) associated with apoptosis, metastatic potential, and matrix metalloproteinases (MMP) from culture supernatants. The 3-D cervical cells infected with *F. gonidiaformans* significantly upregulated expression of the immunosuppressive TNF-related apoptosis-inducing ligand (TRAIL) protein (*p* = 0.0305) (Fig. 2 and Supplementary Fig. 3). The 3-D cervical cells infected with *L. parvula* upregulated expression of the immunosuppressive sFasL (*p* = 0.0240) protein (Fig. 2 and Supplementary Fig. 3). We found no significant differences in the secretion of four MMPs (MMP-1 MMP-7, MMP-9, and MMP-10) in response to all infections, suggesting that MMP secretion by 3-D cervical cells may be mediated by alternative mechanisms (Supplementary Fig. 4).

### Untargeted metabolomics reveals global alterations in amino acid, lipid, and nucleotide metabolism in 3-D cervical cell cultures infected with BVAB

**Fig. 3:**
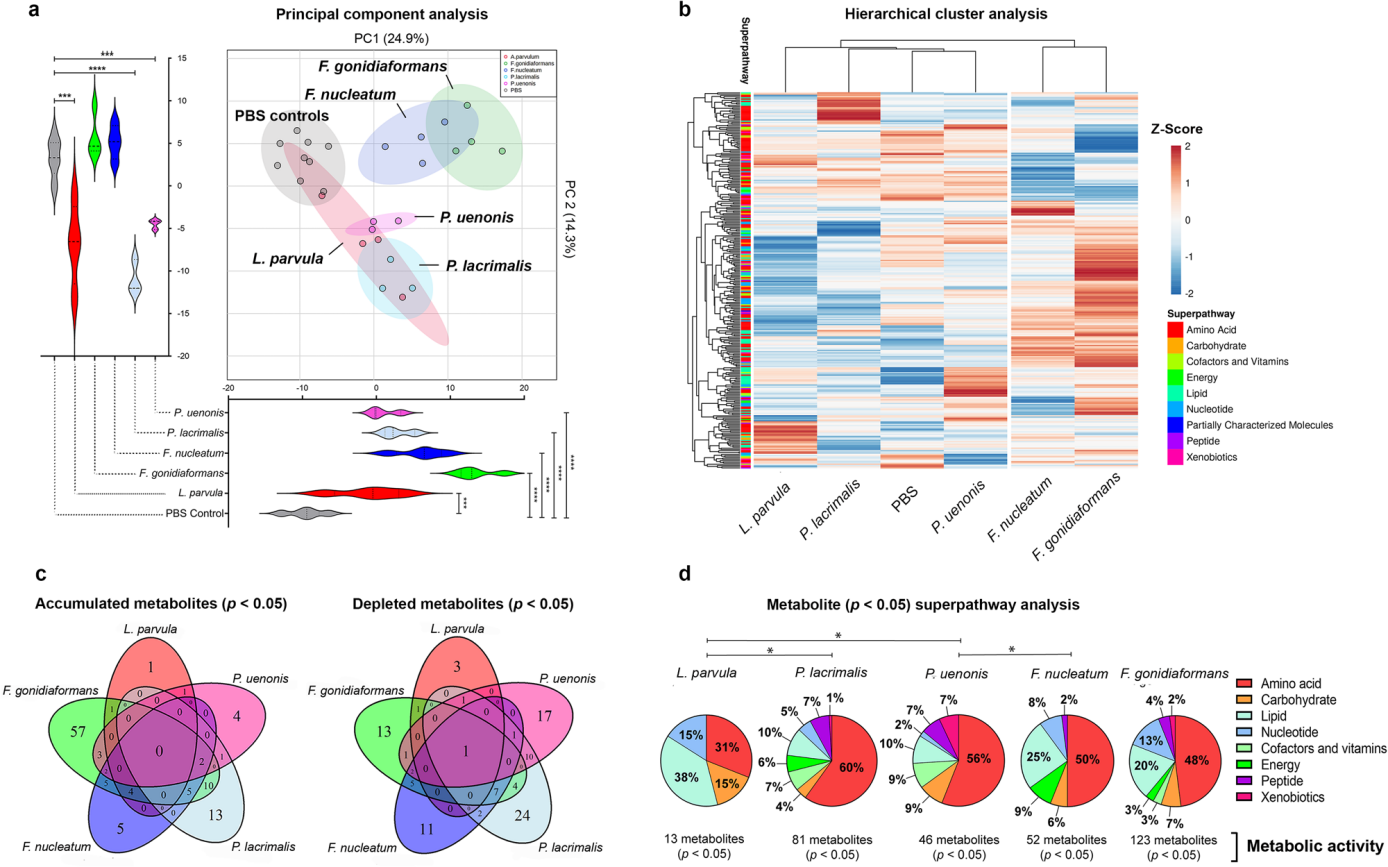
BV-associated bacteria (BVAB) induce distinct metabolomic profiles from mock-infected controls and immunomodulatory signatures of lipids and amino acids. **a** Principal component analysis (PCA) demonstrates that *F. nucleatum* and *F. gonidiaformans* induce similar and partially overlapping metabolic profiles that cluster separately from mock-infected controls. PC1 and PC2 scores were analyzed by unpaired two-tailed Student’s *t*-tests. ***p < 0.001; ****p < 0.0001. **b** Hierarchical cluster analysis (HCA) of metabolite *Z*-scores from 3-D cervical cells infected with BVAB and mock-infected controls. HCA was performed using Euclidean distance and average linkage clustering on both rows (metabolites) and columns (treatments). **c** Venn diagrams indicating the number of unique or overlapping significantly (*p* < 0.05) accumulated or depleted metabolites (infection vs. mock-infected controls). **d** Pie charts represent the percent of significantly different (*p* < 0.05) metabolites (infection vs. mock-infected controls) relative to all metabolites in the superpathway (right). *p < 0.05; Chi-squared (χ^2^) analysis. Infection with BVAB induces global changes in metabolites corresponding to amino acid and lipid superpathways. All bacteria were processed in PBS and adjusted to an optical density at 600 nm (OD_600_) of 0.5. Human 3-D cervical cells were infected with bacterial suspensions (20 μl per 1 × 10^5^ epithelial cells) for 24 h under anaerobic conditions. A minimum of *n* = 3 independent replicates were performed and measured for each condition. Cell culture supernatants were used for global metabolomics analysis.

To gain insights into how BVAB associated with gynecologic cancer modulate the metabolic landscape, we performed untargeted metabolomics on culture supernatants collected from 3-D cervical cells infected with *L. parvula, F. gonidiaformans, F. nucleatum, P. lacrimalis*, or *P. uenonis*. Using liquid chromatography-mass spectrometry, we identified a total of 314 metabolites with a known identity. Metabolic profiles of BVAB were distinct from mock-infected controls, as visualized by principal component analysis (PCA) (Fig. 3a). Principal component 1 (PC1) explained 24.9% of the total variance and all infections were significantly (*p* < 0.001) separated from mock-infected controls. PC2 explained 14.3% of variance and infection with *L. parvula, P. lacrimalis*, and *P. uenonis* significantly (*p* < 0.001) separated from mock-infected controls. Unsupervised HCA grouped *F. gonidiaformans* and *F. nucleatum* into a separate cluster from all other samples (Fig. 3b), suggesting these species modulate metabolomes in a similar manner.

We next analyzed unique and shared metabolites among metabolic profiles of tested BVAB. Differential metabolite abundance was classified as accumulated (fold change >2) or depleted (fold change <0.5) between individual BVAB relative to mock-infected controls. No metabolite was significantly accumulated across all infectious treatments and only one metabolite (5-methylthioadenosine) was significantly depleted in all infections (Fig. 3c). *F. gonidiaformans* and *F. nucleatum* shared the greatest number of identical accumulated (18 total) and depleted (15 total) metabolites relative to mock-infected controls. This suggests genus-specific metabolic activity between both *Fusobacterium* species. Here, we define metabolic activity relative to the number of metabolites that were significantly (*p* < 0.05) different between BVAB and mock-infected controls. *F. gonidiaformans* exerted the greatest metabolic activity (123 altered metabolites) while infection with *L. parvula* exerted the least metabolic activity (13 metabolites) (Fig. 3d). *P. lacrimalis* (81 metabolites)*, P. uenonis* (46 altered metabolites), and *F. nucleatum* (52 altered metabolites) all exhibited intermediate metabolic activity. Metabolites that were significantly altered by BVAB were then grouped by superpathway profiles (Fig. 3d). Superpathway analysis revealed significant differences between *L. parvula* and *P. lacrimalis* (*p* = 0.0159), *L. parvula* and *P. uenonis* (*p* = 0.0494), and between *P. uenonis* and *F. nucleatum* (*p* = 0.0111). Metabolites belonging to amino acid and lipid superpathways were substantially influenced by all BVAB tested. The global results of our untargeted metabolomics data suggest that the BVAB tested in this study modulates the in vitro metabolic extracellular microenvironment in a species- and genus-specific manner.

### Random Forest analysis reveals the oncometabolite 2-hydroxyglutarate and metabolites associated with histidine degradation differentiate BVAB infections

**Fig. 4:**
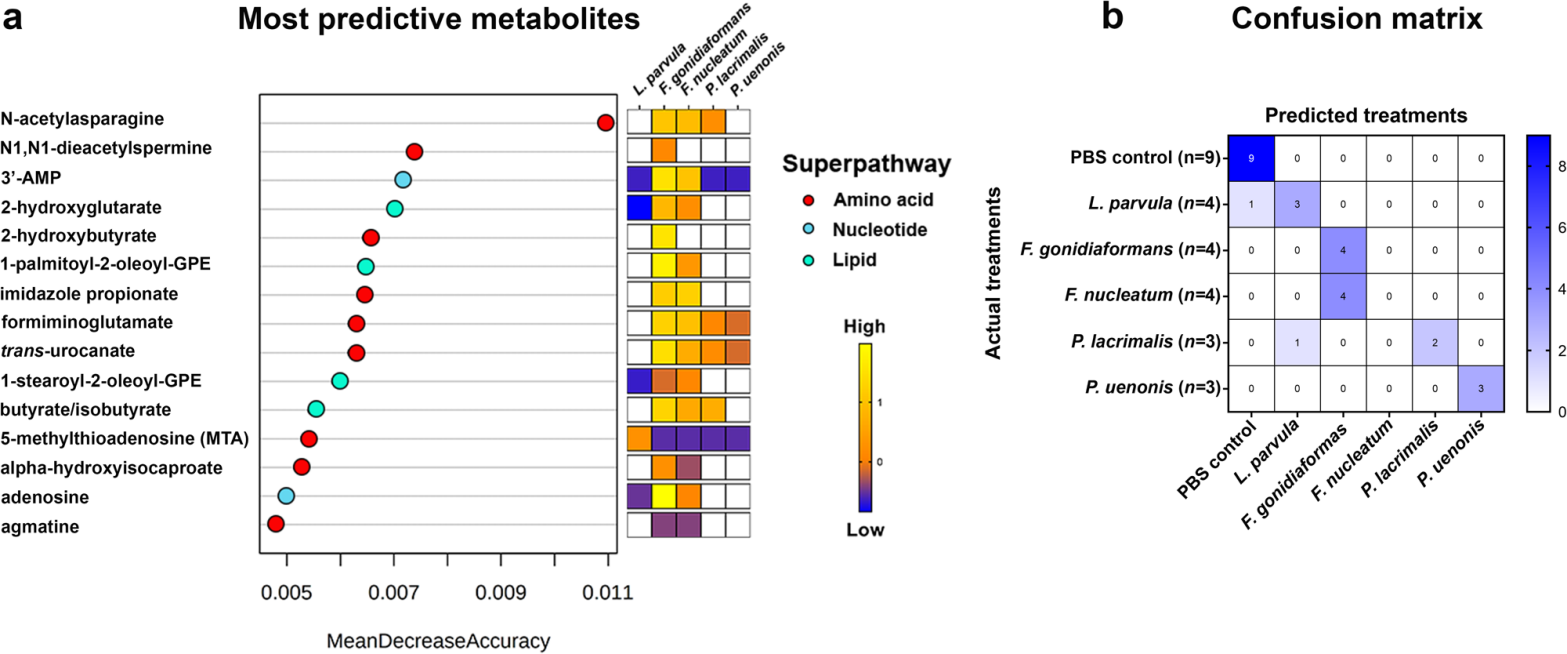
Random Forest classification identifies metabolites within amino acid, nucleotide, and lipid superpathways that discriminate infections with BV-associated bacteria in 3-D cervical cells. **a** Metabolites most predictive of infection with *L. parvula, F. gonidiaformans, F. nucleatum, P. lacrimalis*, and *P. uenonis* include acetylated amino acid derivatives (*N*-acetylasparagine and *N*1,*N*1-diacetylspermine), histidine catabolites (imidazole propionate, formiminoglutamate, and *trans*-urocanate), adenosine derivatives (3’-AMP and adenosine), glycerophospholipids (1-palmitoyl-2-oleyol-GPE and 1-stearoyl-2-oleoyl-GPE), oncometabolite (2-hydroxyglutarate), polyamines (MTA and agmatine), and short-chain fatty acids (2-hydroxybutyrate, butyrate/isobutyrate, and alpha-hydroxyisocaproate). Only significantly different metabolites (infection vs. mock-infected controls) are colored in the heat map. **b** Random Forest confusion matrix. Individual cells are labeled according to how the algorithm predicted each treatment. Uninfected mock controls and infection with *F. gonidiaformans, F. nucleatum*, and *P. uenonis* were perfectly predicted. The number of replicates (*n*) included for each treatment are indicated in parentheses on the vertical axis labels.

**Fig. 5:**
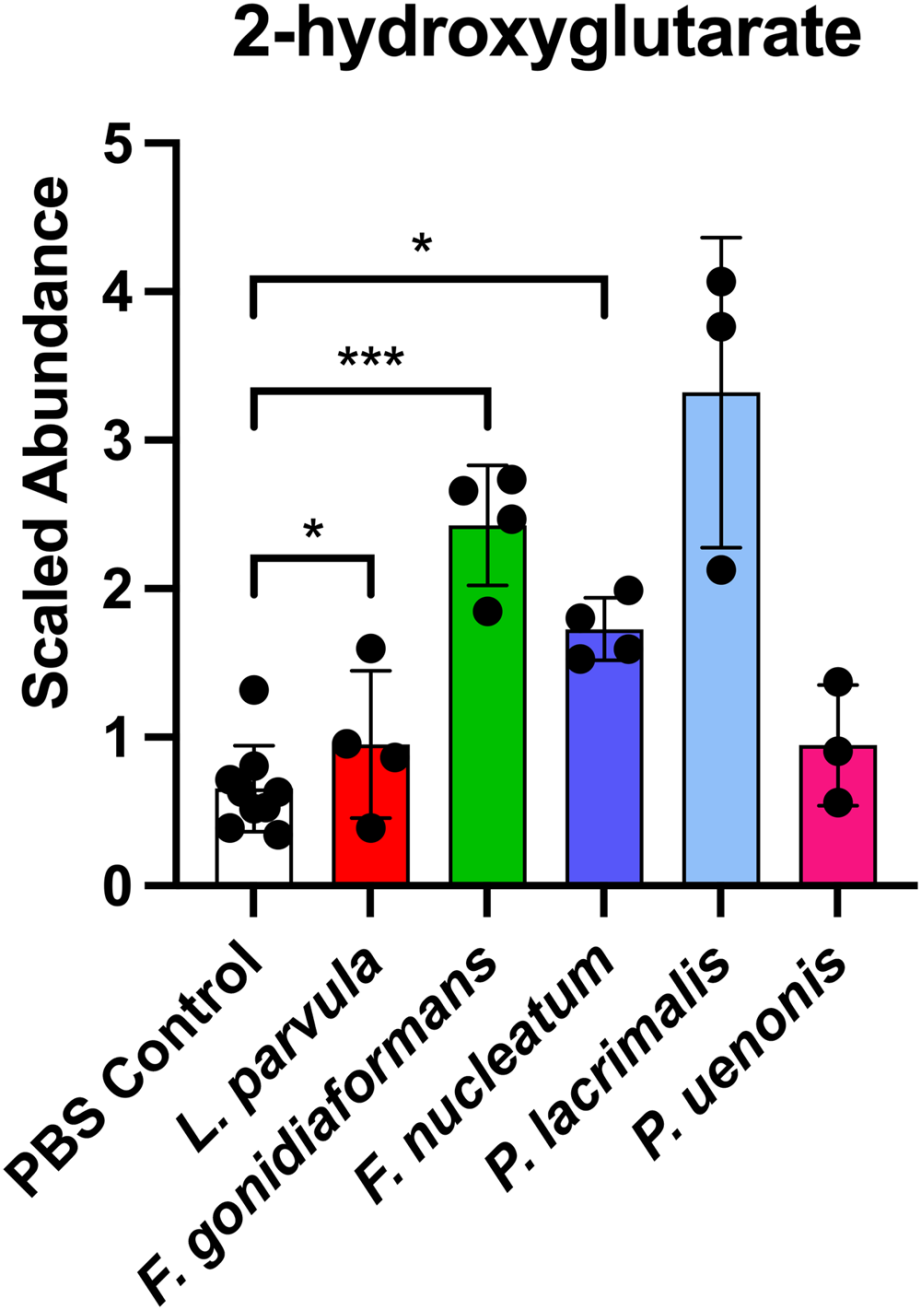
Extracellular supernatants of 3-D cervical cells infected with *L. parvula, F. gonidiaformans*, and *F. nucleatum* infections accumulate the oncometabolite, 2-hydroxyglutarate. Scaled metabolite abundances of 2-hydroxyglutarate after 24 h infection with BVAB. *p < 0.05; ***p < 0.001. Student’s two-tailed paired *t*-tests. Error bars represent standard deviation. A minimum of *n* = 3 independent replicates were performed and measured for each condition.

To predict metabolites that differentiate infection with specific BVAB, we analyzed global untargeted metabolomes using Random Forest (RF) classification algorithms. The most predictive metabolites that differentiate infections belonged to the amino acid, nucleotide, and lipid superpathways (Fig. 4a). Interestingly, three of the top 15 predictive metabolites (imidazole propionate, formiminoglutamate, and *trans*-urocanate) were histidine catabolites. Additionally, the oncometabolite, 2-hydroxyglutarate^25^, was identified by the RF algorithm and was significantly accumulated following infection with *L. parvula, F. gonidiaformans*, and *F. nucleatum* (Fig. 5). These results provide additional evidence for the putative mechanisms by which these BVAB, and possibly other bacteria, may utilize to promote gynecologic carcinogenesis.

Random Forest analyses accurately (100%) predicted infection with *F. gonidiaformans, P. uenonis*, and all mock-infected control samples (Fig. 4b). In contrast, all *F. nucleatum* samples were incorrectly classified as *F. gonidiaformans*, suggesting strong metabolomic signatures shared between the *Fusobacterium* species tested. One *L. parvula* sample was incorrectly predicted as a mock-infected control, which may reflect the relatively low metabolic activity observed for this species. Using global untargeted metabolomics datasets, the RF classification algorithm could accurately discriminate between BVAB infections and identified metabolites that may influence a tumor-promoting cervicovaginal microenvironment.

### BVAB modulates metabolic profiles indicative of histidine degradation

**Fig. 6:**
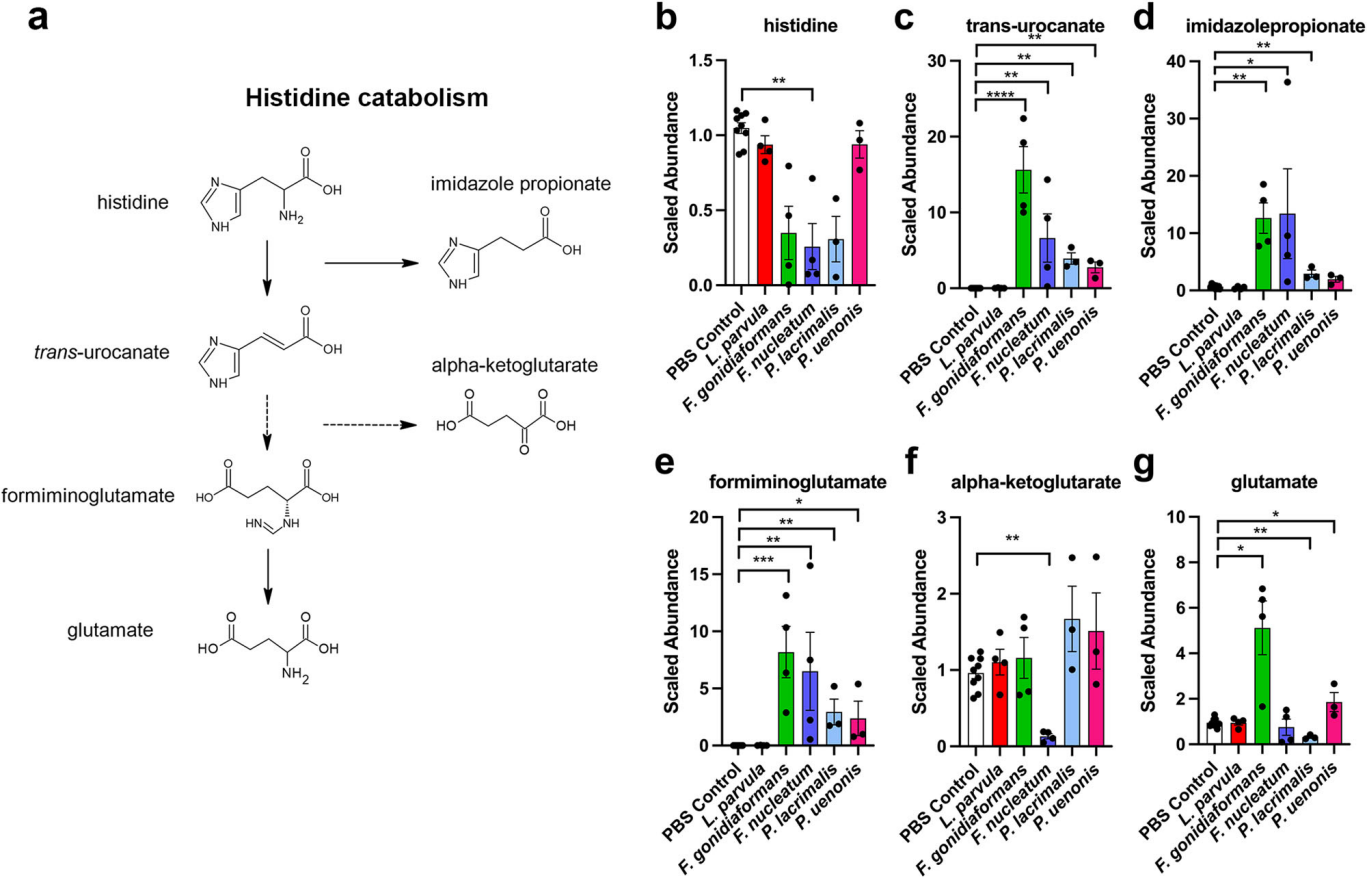
BVAB modulates metabolic profiles corresponding to histidine degradation. **a** Select metabolites of the histidine degradation pathway (KEGG, M00045) corresponding to significantly accumulated or depleted metabolites in the graphs on the right. Graphs represent scaled metabolite abundances of **b** histidine, **c** *trans*-urocanate, **d** imidazole propionate, **e** formiminoglutmate, **f** alpha-ketoglutarate, and **g** glutamate. *p < 0.05; **p < 0.01; ***p < 0.001; ****p < 0.0001. Two-tailed paired Student’s *t*-tests (infection vs. mock-infected controls). Box and whiskers bars show median and error bars represent standard error. A minimum of *n* = 3 independent replicates were performed and measured for each condition.

Three of the predictive metabolites identified by RF (imidazole propionate, formiminoglutamate, and *trans*-urocanate) are metabolites of the histidine catabolism pathway^26^ (Fig. 6). Each of these metabolites were significantly accumulated by infection with at least three BVAB relative to mock-infected controls. Histidine was depleted by infection with *F. gonidiaformans, F. nucleatum*, and *P. lacrimalis*, although the results reached significance only between *F. nucleatum* and mock-infected controls. In regard to other metabolites belonging to the histidine catabolic pathway, α-ketoglutarate (a derivative of the sequential breakdown of *trans-*urocanate^27^) was significantly depleted following infection with *F. nucleatum* (*p* = 0.002). Additionally, the formiminoglutamate catabolite, glutamate^27^, was significantly (*p* < 0.05) elevated following infection with *F. gonidiaformans* and *P. uenonis*. These results suggest that the BVAB tested in this study modulate histidine degradative pathways in 3-D cervical cells^28,29^.

### BVAB modulates extracellular metabolomic profiles corresponding to enrichment in cysteine and methionine, glutathione, and lipid metabolic pathways

To better understand the metabolic networks modulated by BVAB, we performed metabolic pathway enrichment analysis using datasets from individual BVAB relative to mock-infected controls. The KEGG database was used as a reference for metabolic pathway queries. A total of 41 metabolic pathways were significantly (*p* < 0.05) enriched between all comparisons, 13 of which participate in amino acid metabolism and four pathways participating in lipid metabolism. The cysteine and methionine metabolic pathway (KEGG entry hsa00270) was the only pathway significantly enriched in all infections (Supplementary Fig. 5). Butanoate metabolism (KEGG entry hsa00650) and arginine biosynthesis (KEGG entry hsa00220) were highly enriched by infection with *F. gonidiaformans* (6.71-fold enrichment)*, F. nucleatum* (6.67-fold enrichment), and *P. lacrimalis* (3.78-fold enrichment)*. F. gonidiaformans, F. nucleatum,* and *P. lacrimalis* are known butyrate producers^30,31^, thus supporting our data. Metabolic pathways for glycerolipid metabolism (KEGG entry hsa00561) and sphingolipid metabolism (KEGG entry has00600) were also significantly (*p* < 0.05) enriched by infection with *F. gonidiaformans*. Glycerolipids and sphingolipids modulate inflammation and participate in carcinogenic signaling pathways and metastasis^32,33^. The metabolic pathway enrichment analysis supported the metabolic superpathway analyses (Fig. 3d) and provides insights into how BVAB associated with cancer may influence carcinogenesis.

### *F. gonidiaformans* and *F. nucleatum* induce metabolic signatures corresponding to hydrogen sulfide production and oxidative stress

**Fig. 7:**
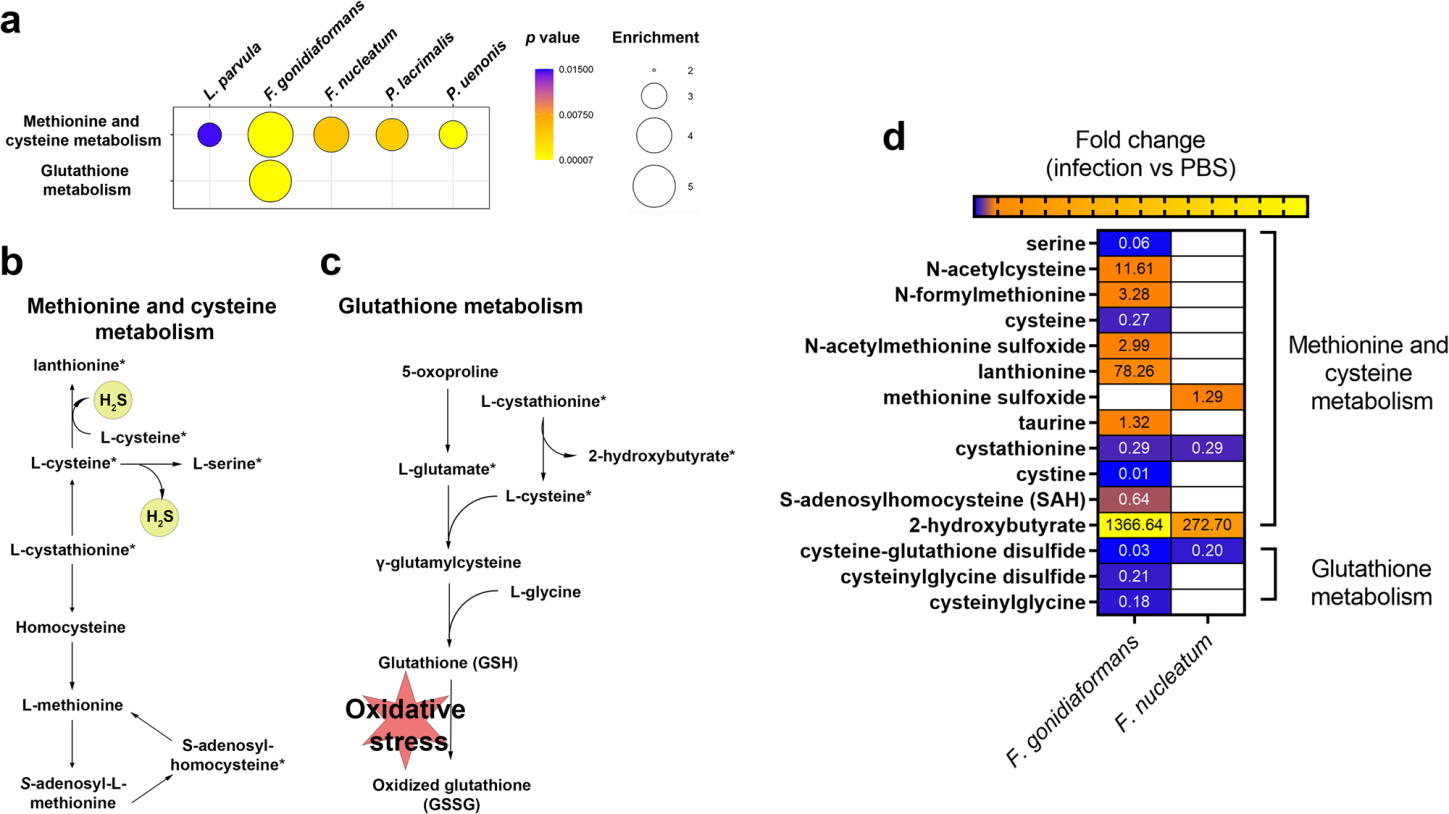
*Fusobacterium* spp. modulate metabolites associated with hydrogen sulfide production and oxidative stress. **a** Bubble plots representing enrichment of methionine and cysteine metabolism and glutathione metabolism from metabolomics data derived from 3-D cervical cells infected with BVAB. Bubble sizes are proportional to the enrichment factor and bubble colors indicate significance. **b** Methionine and cysteine metabolic pathway depicting hydrogen sulfide production (yellow circles). **c** Glutathione metabolic pathway that participates in the oxidative stress response. Asterisks next to metabolites are also represented in the heatmap. **d** Heatmap representing statistically different (*p* < 0.05) metabolites corresponding to methionine and cysteine metabolism and glutathione metabolism that were differentially accumulated (orange and yellow shading) or depleted (blue shading) during infection with *Fusobacterium* species. Fold change difference between infection with and mock-infected controls are indicated as numerical values within the heat map cells. A minimum of *n* = 3 independent replicates were performed and measured for each condition.

The cysteine and methionine metabolic pathway was significantly enriched by infection with all BVAB tested (Fig. 7a and Supplementary Fig. 5): *L. parvula* (2.84-fold; *p* = 0.015), *F. gonidiaformans* (5.47-fold; *p* < 0.0001), *F. nucleatum* (4.03-fold; *p* = 0.005), *P. lacrimalis* (3.67-fold; *p* = 0.004), and *P. uenonis* (3.24-fold; *p* < 0.0001), respectively (Supplementary Fig. 5). The glutathione metabolic pathway was also significantly (*p* < 0.0001) enriched following infection with *F. gonidiaformans* (4.94-fold enrichment) (Fig. 7a). The cysteine and methionine metabolic pathway (Fig. 7b) intersects with the glutathione metabolic pathway (Fig. 7c), the latter of which participates in oxidative stress responses. Both pathways share important metabolic intermediates. We searched our metabolomics datasets for differential accumulation or depletion of specific metabolites belonging to both the cysteine and methionine pathways and glutathione metabolism pathway. Infection with *F. gonidiaformans* and *F. nucleatum* induced differential abundance of metabolites associated with hydrogen sulfide production and oxidative stress. Hydrogen sulfide is generated by lanthionine biosynthesis, the latter of which was significantly accumulated by 78.26-fold by infection with *F. gonidiaformans* relative to mock-infected controls (Fig. 7d). Cysteine desulfuration generates serine and both were significantly (*p* < 0.05) depleted by infection with *F. gonidiaformans*, suggesting high flux through this pathway and hydrogen sulfide production. Increased metabolic flux through the glutathione pathway may suggest increased oxidative stress in the presence of *Fusobacterium* spp., while accumulation of hydrogen sulfide may predispose to host cell genotoxicity^34^.

### *Fusobacterium* spp. infections influence lipid metabolic profiles in 3-D cervical cell cultures

**Fig. 8:**
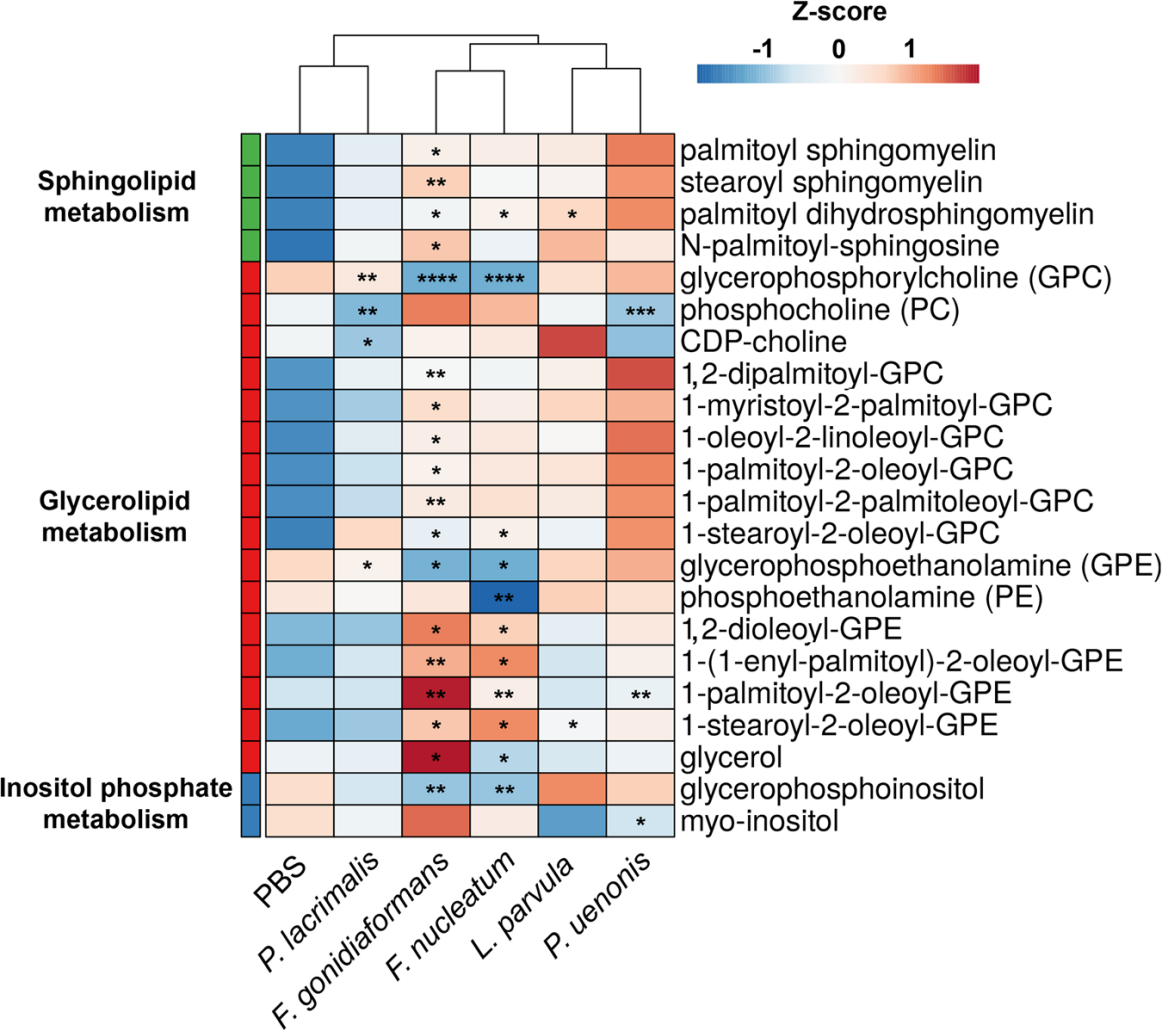
*Fusobacterium* spp. modulate glycerolipid and sphingolipid metabolism during 3-D cervical cell infection. Heatmap of extracellular sphingolipids, glycerolipids, and inositol phosphate metabolites differentially regulated by infection with BVAB. Metabolite intensity values were log-transformed, autoscaled, and row-centered prior to hierarchical clustering analysis using Euclidean distance measures and average linkage clustering algorithms. Asterisks indicate statistically different metabolites between infectious treatments and mock-infected controls. Only lipid species significantly different between at least on BVAB species and mock-infected controls are shown. *p < 0.05; **p < 0.01; ***p < 0.001; ****p < 0.0001. Two-tailed paired Student’s *t*-tests. A minimum of *n* = 3 independent replicates were performed and measured for each condition.

The global metabolomic profiles representing the BVAB tested in this study were enriched in metabolic pathways corresponding to glycerolipid metabolism, sphingolipid metabolism, and inositol phosphate metabolism (Supplementary Fig. 5). We, therefore, investigated the specific lipids that were significantly (*p* < 0.05) accumulated or depleted during infection with BVAB relative to mock-infected controls. Both *Fusobacterium* species elicited the greatest impact on differential lipid abundance. Infection with *F. gonidiaformans* and *F. nucleatum* resulted in alteration of 18 and 11 lipid species, respectively (Fig. 8). HCA analysis grouped *F. gonidiaformans* and *F. nucleatum*, suggesting these species may modulate lipid profiles in a similar manner (Fig. 8). *F. gonidiaformans* induced significant (*p* < 0.05) accumulation of several glycerophosphocholine (GPC) lipids (1-myristoyl-2-palmitoyl-GPC, 1-palmitoyl-2-palmitoleoyl-GPC, 1,2-dipalmitoyl-GPC, 1-palmitoyl-2-oleoyl-GPC, 1-oleoyl-2-linoleoyl-GPC) while both *Fusobacterium* spp. induced significant (*p* < 0.05) accumulation of 1-stearoyl-2-oleoyl-GPC (Fig. 8). Both *Fusobacterium* spp. also induced significant (*p* < 0.05) accumulation of three glycerophosphoethanolamine (GPE) lipids (1-stearoyl-2-oleoyl-GPE, 1-palmitoyl-2-oleoyl-GPE, and 1,2-dioleoyl-GPE). In support of this data, the GPE and GPC head groups corresponding to these lipids were significantly depleted during infection with *Fusobacterium* spp. *L. parvula* and *P. uenonis* clustered together and while these species generally induced glycerolipid and sphingolipid accumulation, only accumulation of palmitoyl dihydrosphingomyelin by *L. parvula* reached significance (*p* = 0.049) relative to mock-infected controls. *P. lacrimalis* grouped with mock-infected controls, suggesting this species does not regulate lipid metabolism to a significant extent. In summary, infection of 3-D cervical cells with *Fusobacterium* spp. resulted in the greatest influence on the differential abundance of sphingolipids and glycerolipids. Accumulation of extracellular lipids may suggest disruption of host cellular membranes, which reflect a compromised cervical epithelial barrier. These data may bear important implications related to how BVAB may generate a pro-inflammatory microenvironment and impact tumor-promoting signaling via alterations of lipid metabolism in the context of the human cervix.

## Discussion

Hanahan and Weinberg initially described the hallmarks of cancer that influence the development and progression of cancerous lesions^35,36^. Hallmarks of cancer in the local microenvironment, such as inflammation, immunosuppression, and pro-oncogenic metabolites might also be influenced by the composition of the resident microbiota^6,24^. For example, *F. nucleatum* and *H. pylori* induce chronic inflammatory host responses that are implicated in the development of colorectal and gastric cancers, respectively^1,37^. *P. gingivalis* exerts immunosuppressive activity that downregulates host adaptive immune functions and may impair antitumoral clearance mechanisms^38^. *Escherichia coli* produces the oncometabolite, 2-hydroxyglutarate, thus potentially implicating this common member of the gut microbiota in colorectal carcinogenesis^39^. Defining the tumor-promoting mechanisms exploited by specific bacterial species at less studied anatomical sites, such as the FRT, is needed to fully understand how dysbiosis of the human microbiome promotes a carcinogenic microenvironment.

In this study, we explored the potential tumor-promoting immunometabolic mechanisms exploited by *L. parvula, F. gonidiaformans, F. nucleatum, P. lacrimalis*, and *P. uenonis*. These BVAB have been associated with malignancy in the FRT and other anatomical sites^8,12,16,24,40^. *F. gonidiaformans* is closely related to *F. necrophorum*^41^ and has been recovered from women with BV^42^. *L. parvula* is related to the commonly isolated BVAB, *F. vaginae*^7^, the latter of which is associated with gynecologic cancers and inflammation^16,20,43^. Using SEM, we demonstrated that *L. parvula, F. gonidiaformans*, and *F. nucleatum* efficiently colonized 3-D cervical cells while *P. lacrimalis* and *P. uenonis* colonized less successfully (Fig. 1). Since BV is a polymicrobial infection, it is possible that *P. lacrimalis* and *P. uenonis* require early colonizers to efficiently adhere to the cervical epithelium. Our group and others hypothesize that cervicovaginal biofilms are initiated by *Gardnerella* and *Prevotella* species^7^, which provide biofilm scaffolds for secondary colonizers such as *Porphyromonas, Peptoniphilus*, and other BVAB species^44^. We found little evidence of cervical cell cytotoxicity in our 3-D and monolayer cultures, following infection with all species tested. This data suggests that these BVAB are not overtly cytotoxic in vitro, given that 3-D cervical cells are more robust against cytotoxic agents when compared to traditional monolayer cultures^17^.

It is well established that chronic inflammation induced by infectious agents, such as viruses and bacteria, can promote carcinogenesis^36,45^. For example, *F. nucleatum* facilitates colorectal carcinogenesis by upregulating pro-inflammatory cytokines and chemokines, immune cell recruitment, and cancer cell migration^37^. Persistent BV is also associated with chronic inflammation^46^ and is manifested through elevated cervicovaginal cytokines and chemokines, such as IL-1β, IL-6, and IL-8^47^. Chronic genital inflammation may improve HPV access to the basal cells of the cervical transition zone, thereby promoting HPV infection and a risk of the development of cervical neoplasia^48^.

Here, we found that *L. parvula, F. gonidiaformans, F. nucleatum*, and *P. lacrimalis* stimulated a pro-inflammatory innate immune response in 3-D cervical cells corresponding to the upregulated secretion of IL-1β, IL-6, and IL-8 (Fig. 2). These pro-inflammatory cytokines and chemokines are often elevated in cervicovaginal lavages collected from women with BV relative to healthy controls^42,49,50^. Additionally, these markers were also induced in 3-D cervical cells in response to other BVAB or microbial compounds^17,20,22^. These immune mediators exert pro-carcinogenic properties. For example, IL-6 can stimulate angiogenesis^51,52^, inhibit apoptosis^53^, and suppress antitumor immune responses^54^. IL-8 is also frequently upregulated in the context of cervical cancer and promotes cell migration and proliferation in vitro^55^. Clinically, high IL-8 titers correspond to advanced-stage cervical neoplasia and poor survival rates^56^. Our group has demonstrated that human 3-D cervical models upregulate the expression of *IL8* in response to infection with *F. vaginae* and *S. amnii*^20,22^, the latter of which is also associated with cervical cancer^8,12,13^. Vaginal and cervical epithelial cells also secrete chemokines to recruit innate and adaptive immune cells to the local microenvironment and clear infection^17,19,57^. Interestingly, *P. uenonis* significantly downregulated secretion of IP-10, RANTES, fractalkine, and MIP-1β by 3-D cervical cells, therefore suggesting suppression of host chemotactic responses that may serve to evade immune clearance. It will be important in the future to further characterize this phenomenon in vitro and in vivo in the context of immune cell recruitment and inflammation. Overall, using a physiologically relevant 3-D cervical cell model and a reductionist approach, we are beginning to illuminate how specific BVAB stimulate inflammatory and chemotactic host responses to generate a tumor-promoting cervicovaginal microenvironment.

Regarding apoptosis-related proteins, we previously reported that FasL was elevated in cervicovaginal lavages of women with vaginal dysbiosis and cervical cancer^9^. By upregulating sFasL in the cervical microenvironment, *L. parvula* may mediate immune suppression and antitumoral escape mechanisms^58^. *F. gonidiaformans* significantly, yet modestly, upregulated secretion of TRAIL, and cervical cancer cells can develop resistance to TRAIL-induced apoptosis^59,60^. Chronic, low-level secretion of TRAIL may select for TRAIL resistance in neoplastic cervical lesions. *L. parvula* and *F. gonidiaformans* may therefore influence local immune suppression and selection for neoplastic subpopulations.

The first described oncometabolite was 2-hydroxyglutarate, which is an aberrant byproduct of mutant human isocitrate dehydrogenase^25^. Bacteria can also generate 2-hydroxyglutarate through lysine degradative pathways^39^ and glutamate fermentation^61^. Additionally, 2-hydroxyglutarate is elevated in women with BV^62^, vaginal dysbiosis^63^ and in women who practice douching^64^, the latter of which is associated with an increased risk of developing BV^65^. BVAB, such as *Fusobacterium* spp., may therefore promote a neoplastic cervicovaginal microenvironment by upregulating 2-hydroxyglutarate and other oncogenic metabolites.

The important metabolites predictive of infections with tested BVAB also suggested a strong metabolic contribution to histidine degradation (Fig. 4a). The metabolite *trans*-urocanate is a marker of filaggrin degradation, a histidine-rich protein and a keratinocyte-specific marker^66^. Genetic defects in filaggrin predispose women to HPV infection and increased cervical cancer mortality^67,68^. Specific BVAB may compromise the cervical epithelial barrier by promoting filaggrin catabolism, thus improving access to the underlying basal cells of the cervical transition zone to drive HPV infection and, subsequently, cervical carcinogenesis. The mechanisms describing how specific BVAB may compromise the cervical epithelial barrier function in vivo are limited and require further investigations.

All BVAB tested modulated metabolomic profiles that were enriched in the methionine and cysteine metabolic pathway (Fig. 7). Cysteine is an important metabolite that also intersects with glutathione metabolism. Glutathione participates in the detoxification of genotoxic reactive oxygen species^69^ and may become limited during prolonged oxidative stress^70^. Cleavage of l-cystathionine replenishes l-cysteine and generates 2-hydroxybutyrate, the latter of which is a metabolic marker of oxidative stress^71^. Infection with both *Fusobacterium* species induced significant accumulation of 2-hydroxybutyrate and depletion of glutathione intermediates. These metabolic signatures of oxidative stress have been reported in clinical samples collected from women with BV^62^. Our previous clinical study investigating cervicovaginal metabolomes in women with and without cervical neoplasia also showed that cervicovaginal levels of 2-hydroxybutyrate were positively correlated with genital inflammation, *Lactobacillus* depletion, and cervical cancer^72^. Though we did not detect reduced or oxidized glutathione in this study, the differential abundance of other metabolites participating in glutathione metabolism suggests that *Fusobacterium* spp. induces oxidative stress in human 3-D cervical cells. Additional work is needed to confirm whether this phenomenon is mirrored in the clinical setting.

We found signatures of hydrogen sulfide production by *Fusobacterium* spp. (Fig. 7), which is in accordance with previous studies^61,73^. Lanthionine was highly accumulated during infection with both *Fusobacterium* spp. This nonproteinogenic amino acid is a component of the *Fusobacterium* peptidoglycan structure^61,74^. Lanthionine biosynthesis generates hydrogen sulfide, which has been associated in a mouse model with advanced-stage ovarian cancer^75^ and may be genotoxic^34^. Lanthionine biosynthesis may therefore contribute to the tumor-promoting properties of *Fusobacterium* spp. in the cervicovaginal microenvironment^13,76^.

*F. gonidiaformans* and *F. nucleatum* similarly modulated lipid metabolism (Fig. 8). Our data suggest that *F. nucleatum* and *F. gonidiaformans* may exploit host-derived ethanolamine to sustain carbon and nitrogen demands in the cervicovaginal microenvironment^61^. We also found evidence of extracellular sphingolipid accumulation during infection with *F. gonidiaformans, F. nucleatum*, and *L. parvula*. In clinical settings, both sphingomyelins and *Fusobacterium* were found to be elevated in fecal samples collected from colorectal cancer patients relative to healthy controls^77^. *Fusobacterium* spp. and *L. parvula* may therefore modulate a tumor-promoting microenvironment by inducing sphingolipid accumulation in the lower FRT.

In this study, we have provided novel insights into the putative immunometabolic mechanisms employed by understudied BVAB associated with cancer using a well-characterized 3-D human cervical cell model. It is important to study the tumor-promoting mechanisms of these BVAB, despite their low relative abundance in the cervicovaginal microbiome, as such species may still modulate the FRT microenvironment in a manner that provides pro-carcinogenic “second hits” that synergize with other factors to promote the cancerous phenotype. Given that cervical carcinogenesis is largely mediated by HPV, it may be possible that specific BVAB alter the immunometabolic landscape to promote HPV infection and persistence, therefore increasing the likelihood of cervical neoplastic events. Additional studies are needed to determine whether these and other BVAB modulate the immunometabolic landscape in a similar fashion to promote other gynecologic malignancies, such as endometrial cancer^78^.

Limitations of our current study include the lack of modeling of bacterial diversity associated with BV; however, here, we aimed to dissect the individual contributions of specific understudied BVAB to avoid confounding results associated with inter-bacterial interactions. In the context of metabolism, it is likely that some metabolites are generated by one or more bacterial species and utilized by others^79,80^. This may be reflected in the low metabolic activity seen with *L. parvula* and, to a lesser extent, *P. lacrimalis* and *P. uenonis* infections. Such co-interactions may profoundly influence the local metabolic microenvironment and will be the focus of future studies. Additionally, bacterial co-interactions may synergistically modulate the host-pathogen immune response^20^. We cannot exclude the possibility of inter-strain differences corresponding to the species studied here in the context of differential metabolomic and inflammatory responses. Previously, we have determined the immunometabolic impact of health-associated lactobacilli and more common BVAB associated with cervical cancer, such as *F. vaginae* and *S. amnii*, using similar experimental approaches^22^. Though this report lacks a commensal species, such as lactobacilli, the inclusion of PBS mock-infected controls provided in this report allows us to derive immunoproteomic and metabolomic conclusions against healthy (i.e., non-infected) 3-D cervical cells. The strains utilized in this paper are usually present at relatively low levels in women with BV and gynecologic cancers; therefore, it remains unknown whether these species directly influence pro-tumorigenic mechanisms or if their presence in the context of cancer is due to a favorable microenvironment (e.g., nutrient availability due to ulceration and bleeding). Clinical longitudinal studies are required to determine the colonization kinetics of BVAB associated with gynecologic malignancies and whether such species conform to the “driver-passenger” model^81^. In this manner, it is important to differentiate whether other understudied BVAB associated with gynecologic cancer directly promote malignancy (“driver”) or if the cancerous microenvironment (e.g., ulceration and bleeding) favors colonization with these species (“passenger”). The data provided in this report provides the foundations for future studies that incorporate infection with polymicrobial “cocktails” to explore how bacterial co-interactions influence the host defense responses of 3-D cervical cells.

**Fig. 9:**
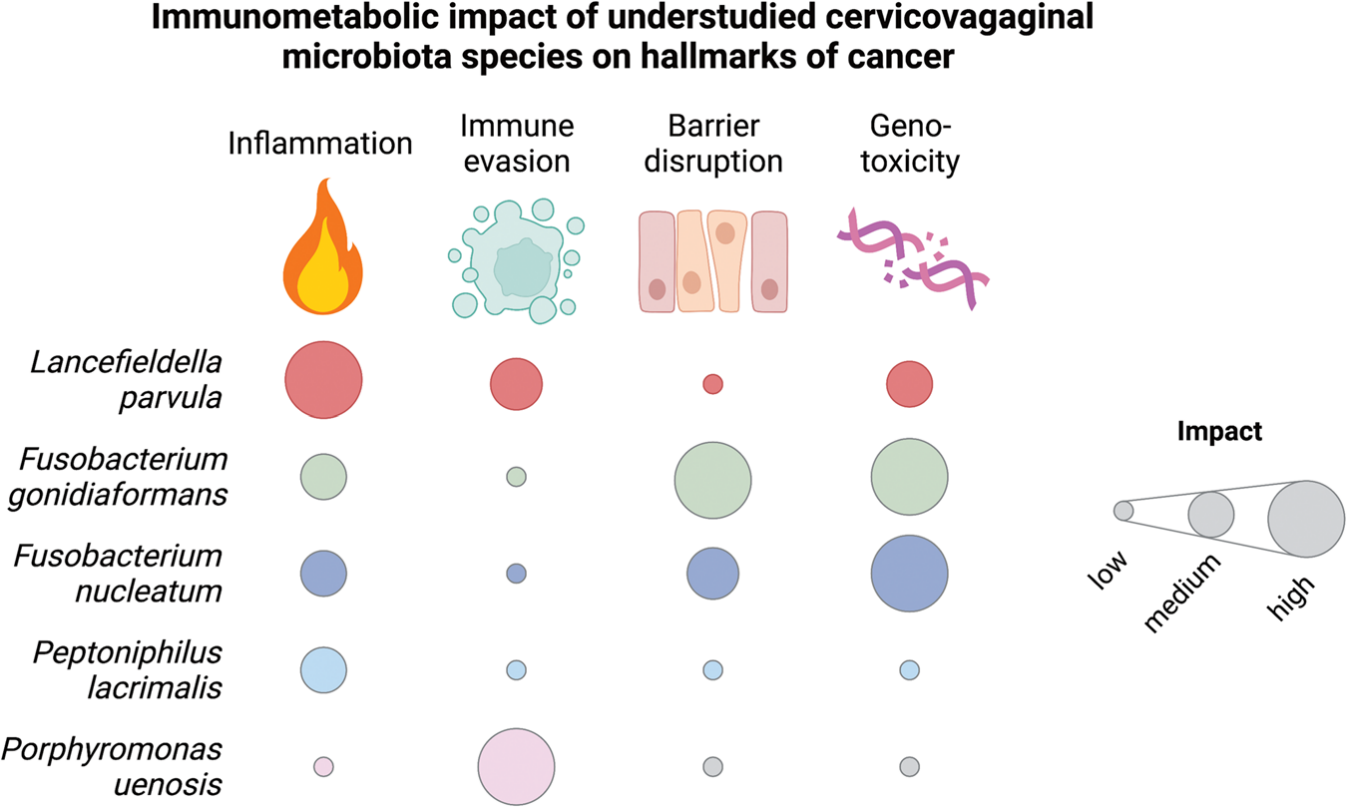
Summary of hallmarks of cancer influenced by BVAB associated with cancer. Heatmap depicts the relative impact that each bacterial species contributes to the putative pro-tumorigenic microenvironment based on our 3-D cervical cell model. Heatmap colors were assigned based on standardized levels of significantly (*p* < 0.05) modulated immunoproteomic and metabolomic biomarkers related to the hallmarks of cancer such as inflammation, avoiding immune destruction, barrier disruption, and genomic instability.

In summary, we identified putative mechanisms by which BVAB associated with various cancers, including gynecologic malignancies, may create a tumor-promoting immunometabolic microenvironment (Fig. 9). Of the BVAB tested, *L. parvula, P. lacrimalis*, and *P. uenonis* influenced the immune host response to the greatest extent while *F. gonidiaformans* and *F. nucleatum* elicited the greatest metabolic activity and modulated metabolic hallmarks of cancer. Additionally, all BVAB tested except *L. parvula* elicited metabolic signatures of histidine catabolism that may be correlated with disruption of the cervical epithelium. In conclusion, this data suggests that particular BVAB may promote HPV infection and persistence and consequently cervical neoplasia by generating pro-inflammatory responses and upregulating metabolic hallmarks of cancer. Our study provides a foundation for further defining the role of understudied BVAB species in promoting a cervicovaginal immunometabolic microenvironment permissive for carcinogenesis.

## Methods

### Human endocervical epithelial cell culture

Human endocervical epithelial A2 cells^82^ were routinely maintained in keratinocyte serum-free medium (KFSM) supplemented with recombinant epidermal growth factor (5 ng/ml), bovine pituitary extract (50 µg/ml; Gibco), CaCl_2_ (22 mg/ml; Sigma), and primocin (100 µg/ml, InvivoGen), herein referred to as A2 medium. Cervical cells were cultured as monolayers or as three-dimensional (3-D) models, as previously described^17,83^, at 37 °C under 5% CO_2_ atmosphere.

### Bacterial strains and culture conditions

*Atopobium parvulum* (recently reclassified as *L. parvula*) strain DNF00906, *Fusobacterium gonidiaformans* strain CMW8396, *F. nucleatum* strain MJR7757B, *P. lacrimalis* strain DNF00528, and *Porphyromonas uenonis* strain UPII 60-3 were used in this study. All strains were obtained from the Biodefense and Emerging Infections (BEI) repository. *L. parvula* and *P. lacrimalis* were cultured on tryptic soy agar (Becton Dickinson) supplemented with 5% defibrinated sheep blood (Quad Five). *F. gonidiaformans*, *F. nucleatum*, and *P. uenonis* were cultured on brain-heart infusion agar supplemented with 5% defibrinated sheep blood. All strains were routinely maintained at 37 °C under anaerobic conditions generated by an AnaeroPack system (Thermo Scientific).

### Bacterial infection of cervical cell monolayers

Cervical cell monolayers were seeded into tissue-treated 24-well plates to a density of ~2 × 10^5^ cells per well and incubated overnight in an antibiotic-free A2 medium at 37 °C under 5% CO_2_ atmosphere. Bacterial strains were sub-cultured onto fresh agar medium at 37 °C under anaerobic conditions 24-h prior to infection. Bacterial strains were harvested and resuspended into sterile Dulbecco’s phosphate-buffered saline (PBS). The bacterial species tested formed residual bacterial aggregates during processing and proved difficult to generate a homogenous cell suspension in PBS. We, therefore, adjusted all species to a standard optical density at 600 nm (OD_600_) of 0.05, 0.5, and 5.0 and cervical cells were infected with adjusted bacterial suspensions (20 μl per 1 × 10^5^ cells). PBS-treated mock infections served as controls. Infected cervical cells were incubated under anaerobic conditions at 37 °C for 24-h prior to use for downstream experimental manipulation. All experiments were performed as three independent biological replicates.

### Bacterial infection of 3-D cervical cell models

The 3-D cervical cell models were differentiated for 28 days in rotating-wall vessel (RWV) bioreactors, as previously described^17,83^. The 3-D cervical cell models were then harvested, washed in pre-warmed antibiotic-free A2 medium, and distributed into tissue-treated 24-well culture plates (1 × 10^5^–5 × 10^5^ cells/ml). Bacteria were processed from agar medium and adjusted to an OD_600_ of 0.5 as described above and used to infect 3-D cervical cells (20 μl per 1 × 10^5^ cells). Infected 3-D cells were incubated under anaerobic conditions at 37 °C. PBS-treated mock infections served as controls. Cell culture supernatants were harvested 24-h post-infection and immediately stored at −80 °C until further use. For scanning electron microscopy (SEM) experiments, 3-D cervical cells were infected with bacterial suspension (OD_600_ of 5.0, 20 μl per 1 × 10^5^ cells) for 4 h. Colonized 3-D cervical cells were fixed in 2.5% glutaraldehyde and visualized by SEM, as previously described^17^. All samples were processed and visualized at the Arizona State University Eyring Materials Center Life Sciences Electron Microscopy Lab using a JEOL JSM 6300 scanning electron microscope.

### Cytotoxicity assays

Cervical epithelial cell monolayers were individually infected with bacteria, as described above. Cervical cell cytotoxicity was measured by trypan blue exclusion using a hemocytometer. All experiments were performed as three independent biological replicates.

### Bio-Plex analyses

Immunoproteomic profiles were determined in cell culture supernatants collected from 3-D cervical cell models infected with bacterial strains and PBS mock-infected controls. Cytokines (interleukin-1α (IL-1α), IL-1β, IL-1RA, IL-6, transforming growth factor-alpha (TGFα), and tumor necrosis factor-alpha (TNFα)), chemokines (IL-8, IP-10, macrophage chemotactic protein-1 (MCP-1), MCP3, MIP-1β, fractalkine, and RANTES), and growth factors (platelet-derived growth factor-AA (PDGF-AA) and vascular endothelial growth factor (VEGF)) were measured using the MILLIPLEX^®^ MAP Human Cytokine/Chemokine Panel 1, Th17 Panel, and Sepsis Panel 2. Matrix metalloproteinases (MMP-1, MMP-7, MMP-9, and MMP-10) were measured using the MILLIPLEX^®^ MAP MMP Panel 2. Circulating cancer biomarkers (macrophage migration inhibitory factor (MIF), TRAIL, sFasL, carcinoembryonic antigen (CEA), and cancer antigen 125 (CA125)) were quantified using MILLIPLEX^®^ MAP Circulating Cancer Biomarker Panel 1. (Millipore, Billerica, MA). Data were collected using a Bio-Plex 200 platform and analyzed using Manager v5.0 software (Bio-Rad, Hercules, CA). A five-parameter logistic regression curve fit was used to determine the concentration. At least three independent biological replicates per experimental condition, each containing two technical replicates, were analyzed.

### Global untargeted metabolomics

Frozen supernatants collected from 3-D cervical cell infections and PBS mock-infected controls were sent to Metabolon Inc. (Durhan, NC) for ultrahigh performance liquid chromatography-mass spectrometry (UPLC-MS) untargeted metabolomics, as previously described^72^. Metabolites were resolved on a Waters ACQUITY UPLC unit and detected using a Thermo Scientific Q-Exactive mass spectrometer interfaced with a heated electrospray ionization (HESI-II) source and Orbitrap mass analyzer operated at 35,000 mass resolution. The MS analysis alternated between MS and data-dependent MS^n^ scans using dynamic exclusion. The scan range varied slighted between methods but covered 70–1000 m/z. Raw mass spectrum data was extracted, peak-identified and QC processed using Metabolon’s Laboratory Information Management System (LIMS). Compounds were identified by comparison to library entries of purified standards or recurrent unknown entities. At least three independent biological replicates per experimental condition were submitted for metabolomics analyses.

### Bioinformatic and statistical analyses

Random Forest classification, Spearman’s correlation analysis, principal component analysis (PCA), and metabolite pathway enrichment were performed using MetaboAnalyst 4.0^84^. Hierarchical clustering analysis (HCA) was performed using the online ClustVis software by Euclidean distance measures and average linkage clustering. Median-centered metabolite peak intensity values were ln(x) transformed and subject to autoscale normalization prior to HCA analysis.

### Statistics and reproducibility

We used two-tailed unpaired Student’s *t*-tests to compare cytotoxicity and Bio-Plex data between infections and untreated mock infections (PBS controls). Metabolite peak intensity values were median-centered and missing values were imputed with half the minimum value across all samples for a given metabolite. Univariate statistical analyses on normalized data (infection vs. PBS control) was performed by two-tailed paired Student’s *t*-tests using the rstatix R package. To correct for multiple comparisons, false discovery rate (FDR) adjusted *p* values (*q* values) were calculated using the *q* value R package. All experiments and statistical analyses, except for SEM, were performed as independent studies with at least three biological replicates. Technical replicates were defined as sample measurements taken from within a single biological replicate.

### Reporting summary

Further information on research design is available in the Nature Research Reporting Summary linked to this article.

## Supplementary information

Supplemental Information

Description of Additional Supplementary Files

Supplementary Data 1

Supplementary Data 2

Reporting Summary

## Data Availability

The authors declare that the data supporting the findings of this study are available within the paper and its Supplementary Files. Source data for all graphs and heatmaps are available in the Supplemental Files.

## Code availability

R scripts used for statistical analysis of metabolomics data are available from the corresponding author upon reasonable request.
