# Supplementary material for: Immunometabolic and potential tumor-promoting changes in 3D cervical cell models infected with bacterial vaginosis-associated bacteria
Source: Commun Biol. 2022 Jul 22;5:725. doi: 10.1038/s42003-022-03681-6 (PMC9307755; doi:10.1038/s42003-022-03681-6)
Supplement: Supplementary file 1 — Supplemental Information [file 42003_2022_3681_MOESM1_ESM.pdf]

Supplementary Information

Immunometabolic and oncogenic potentials of understudied cervicovaginal microbiota related to bacterial vaginosis and gynecologic cancer

Jason D. Maarsingh[1], Paweł Łaniewski[2], and Melissa Herbst-Kralovetz[1,2,*]

[1]Department of Obstetrics and Gynecology, College of Medicine-Phoenix, University of Arizona, Phoenix, AZ, USA

[2]Department of Basic Medical Sciences, College of Medicine-Phoenix, University of Arizona, Phoenix, AZ, USA

* Corresponding author

Email: mherbst1@arizona.edu (MHK)

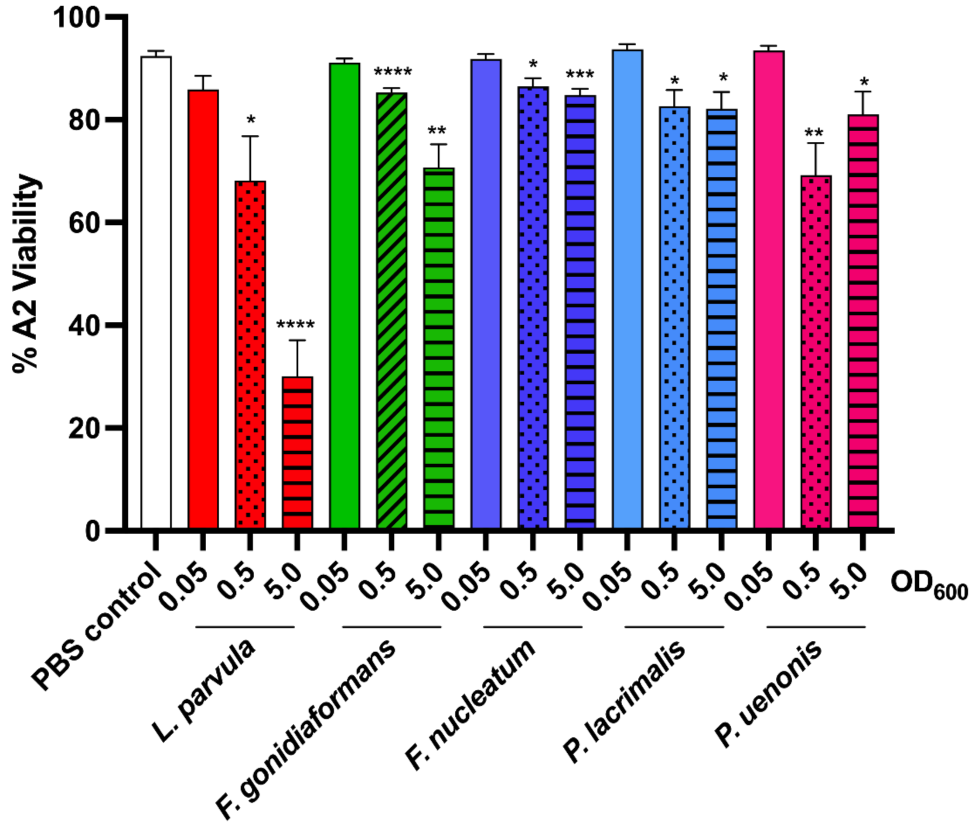

Supplementary Figure 1. BVAB induce modest cytotoxic against cervical cell monolayers.

Trypan blue exclusion staining demonstrating dose-dependent cytotoxicity of *L. parvula, F. gonidiaformans, F. nucleatum, P. lacrimalis,* and *P. uenonis* against cervical cell monolayer cultures. Cervical cells were infected with BVAB at three doses: low, medium, high corresponding to final OD_{600} of 0.1, 0.01, and 0.001 per $1x10^5$ cervical cells/mL, respectively. Infected cultures were incubated anaerobically at 37 ºC for 24 h prior to obtaining experimental results. Error bars represent standard deviation. All experiments were performed as three independent experiments. *, p<0.05; **, p<0.01; ***, p<0.001. One-way ANOVA with Dunnett's multiple comparisons against mock-infected controls.

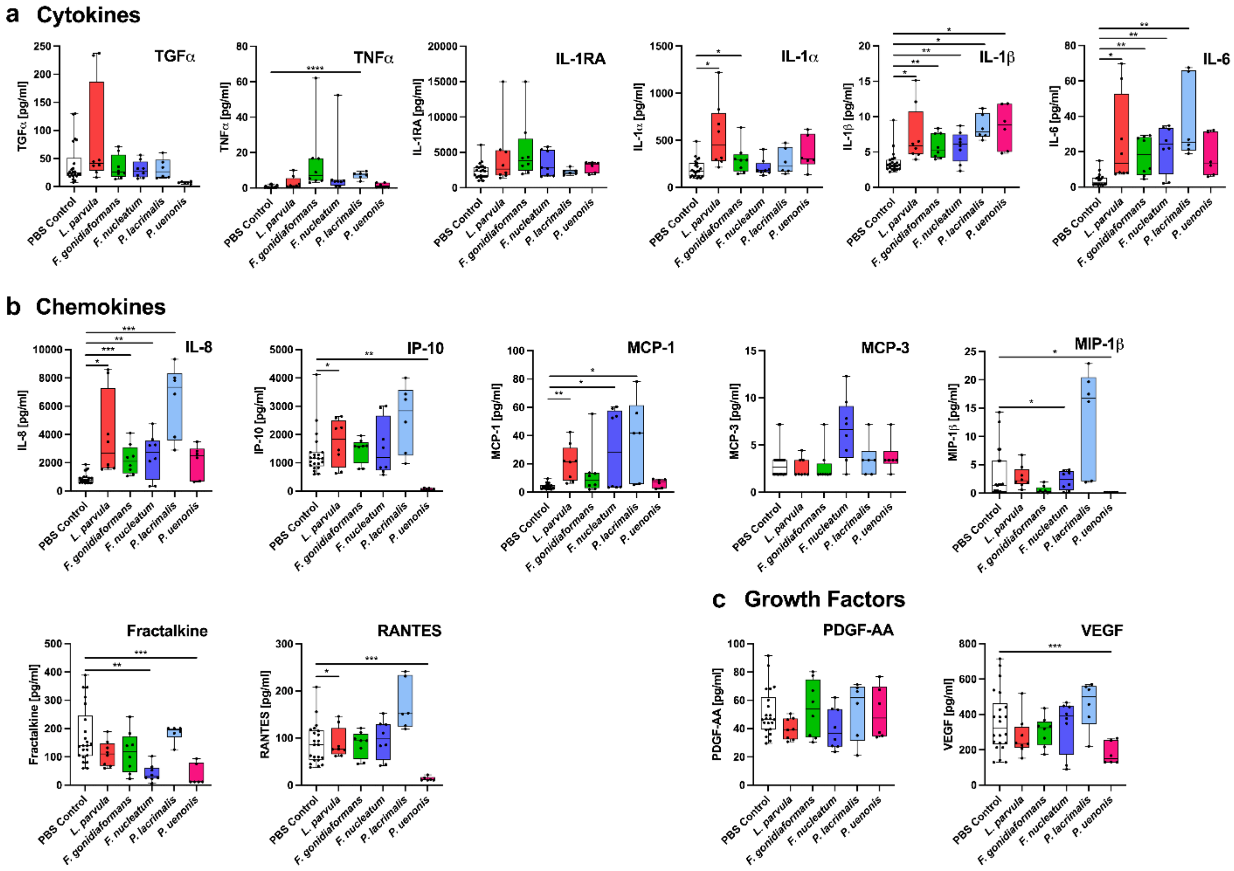

Supplementary Figure 2. BVAB associated with gynecological cancer induce pro-inflammatory cytokine and chemokine responses in 3-D cervical cells.

Bio-Plex analysis of cytokines (A), chemokines (B), and growth factors (C) secreted by 3-D cervical cells infected with BVAB for 24 h. *, $p<0.05$; **, $p<0.01$; ***, $p<0.001$; ****, $p<0.0001$. Two-tailed unpaired Student's t-test (infection vs. mock-infected controls). Error bars represent standard deviation.

Circulating cancer biomarkers

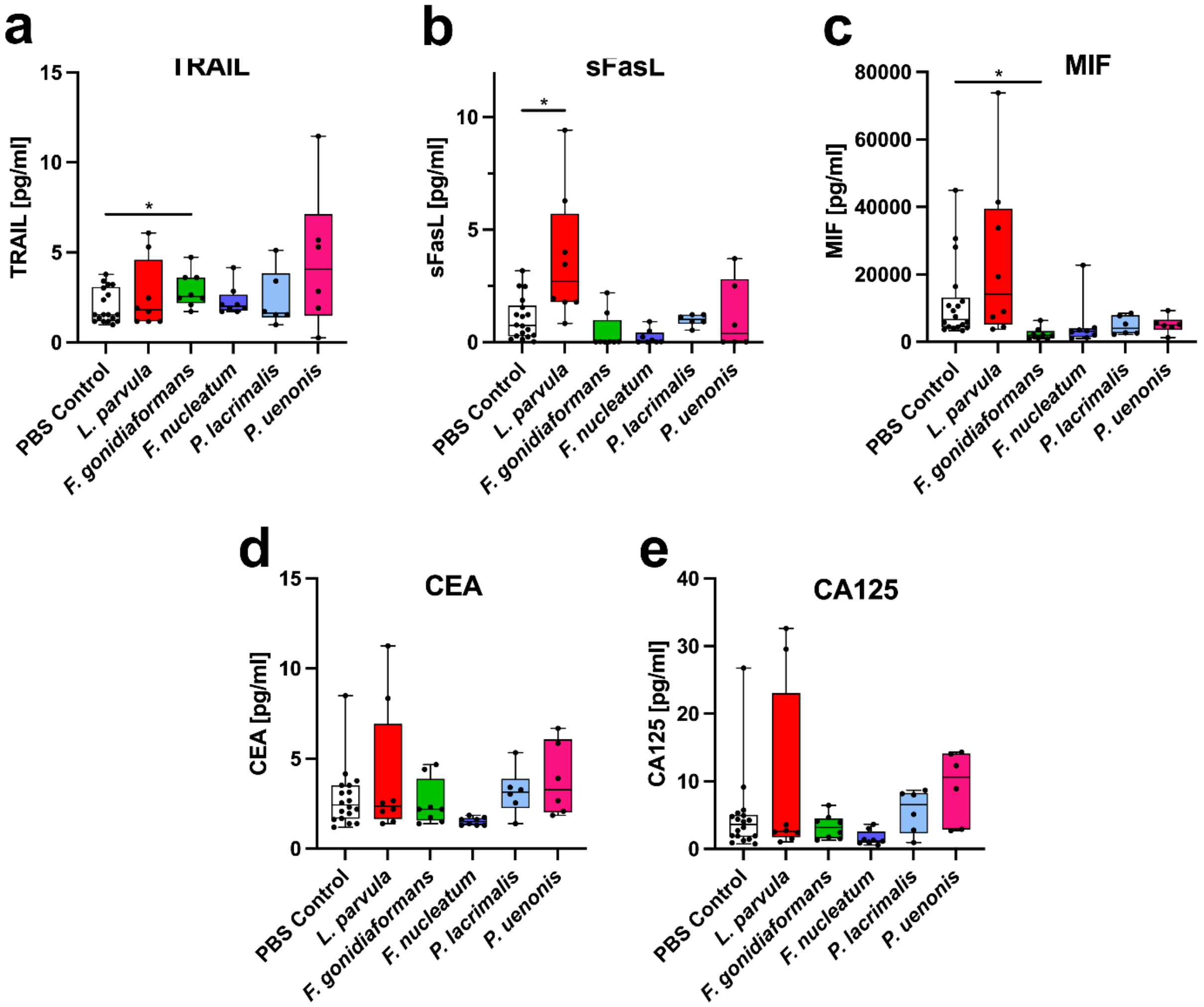

Supplementary Figure 3. *A. parvulum* and *F. gonidiaformans* induce cancer biomarker signatures during 3-D cervical cell infection.

Bio-Plex analysis of circulating cancer biomarkers induced by BVAB for 24 h. *, $p<0.05$. Unpaired two-tailed Student's t-test (infection vs. mock infected controls). Error bars represent standard deviation.

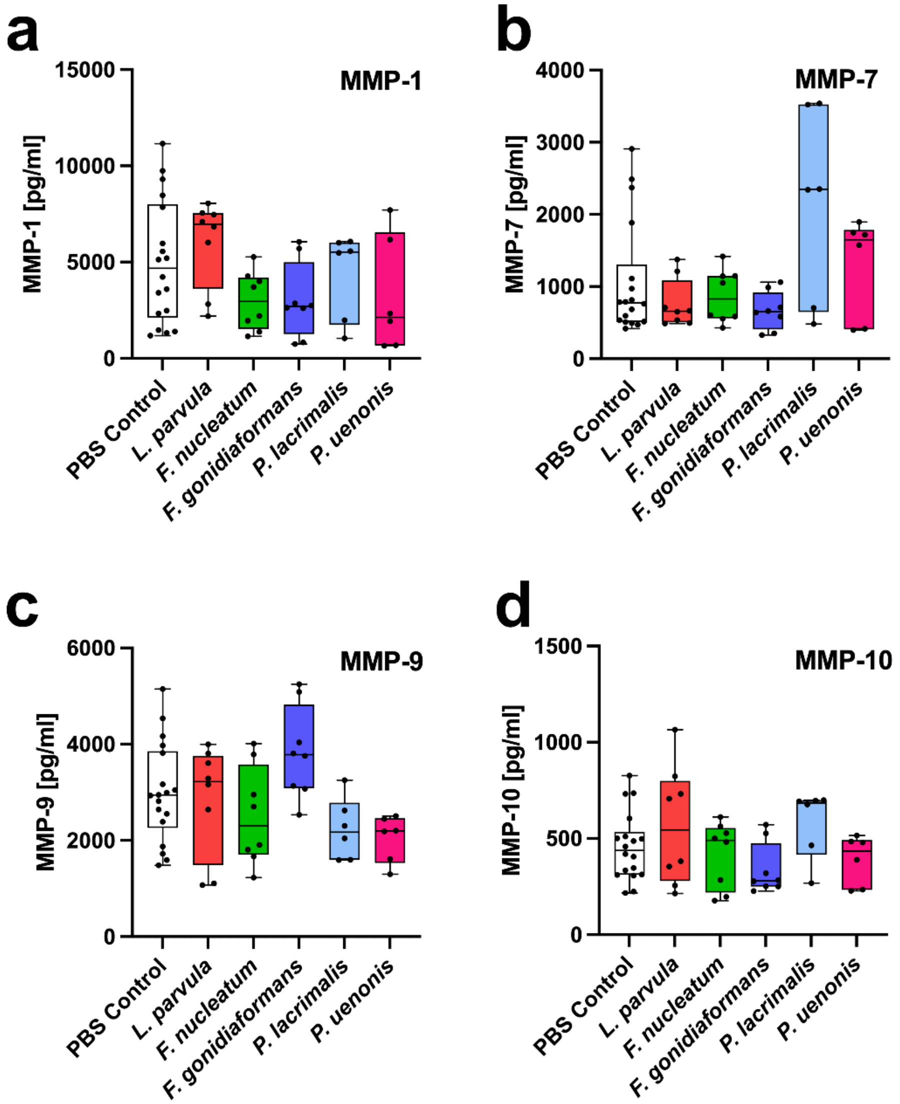

Supplementary Figure 4. BVAB associated with gynecological cancer do not modulate matrix metalloproteinase responses in 3-D cervical cells.

Bio-Plex analysis of matrix metalloproteinases (MMPs) induced by BVAB for 24 h. Unpaired two-tailed Student's t-test (infection vs. mock infected controls). Error bars represent standard deviation.

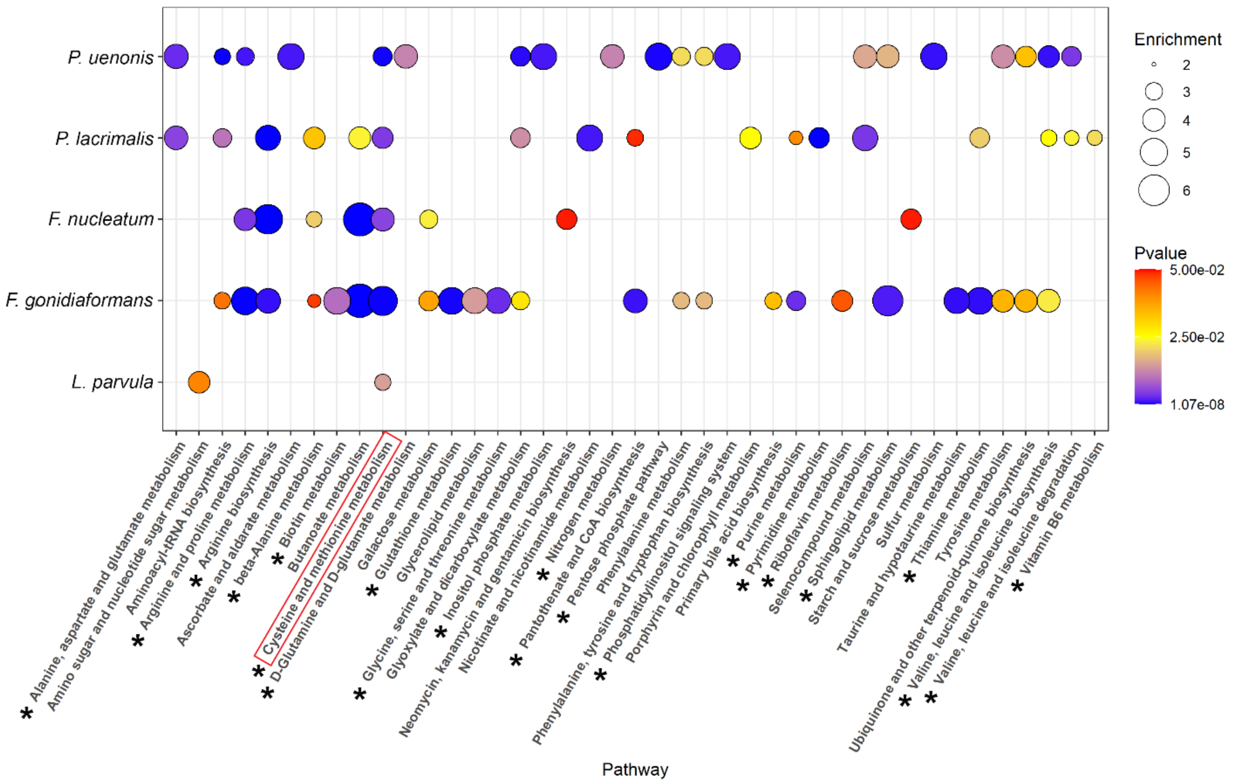

Supplementary Figure 5. BVAB modulate enrichment of metabolic pathways corresponding to amino acid and lipid metabolism.

Bubble plots of significantly enriched KEGG metabolic pathways based on comparisons of metabolomic profiles of infection vs. mock-infected controls. Bubble plot size is proportional to the enrichment value. Bubbles are colored based on significance (*p*-value) of enriched metabolic pathways. Asterisks denote metabolic pathways knowns to be associated with cancer. The cysteine and methionine metabolic pathway (denoted within the red box) was significantly (p<0.05) enriched by all BVAB tested in this study.
