# Supplementary material for: Immunometabolic and potential tumor-promoting changes in 3D cervical cell models infected with bacterial vaginosis-associated bacteria
Source: Commun Biol. 2022 Jul 22;5:725. doi: 10.1038/s42003-022-03681-6 (PMC9307755; doi:10.1038/s42003-022-03681-6)
Supplement: Supplementary file 2 — Description of Additional Supplementary Files [file 42003_2022_3681_MOESM2_ESM.pdf]

Description of Additional Supplementary Files

File name: Supplementary Data 1
Description: Metabolite and pathway list including fold-change (infection vs. PBS control) and statistical (*p* & *q*) values.

File name: Supplementary Data 2

Description: Source Data
